# Contrasting Effects of Nitrogen Addition on Vegetative Phenology in Dry and Wet Years in a Temperate Steppe on the Mongolian Plateau

**DOI:** 10.3389/fpls.2022.861794

**Published:** 2022-04-25

**Authors:** Zhenxing Zhou, Liwei Zhang, Yinzhan Liu, Kunpeng Zhang, Wenrui Wang, Junkang Zhu, Shijie Chai, Huiying Zhang, Yuan Miao

**Affiliations:** ^1^International Joint Research Laboratory for Global Change Ecology, School of Life Sciences, Henan University, Kaifeng, China; ^2^School of Biological and Food Engineering, Anyang Institute of Technology, Anyang, China; ^3^Taihang Mountain Forest Pests Observation and Research Station of Henan Province, Linzhou, China

**Keywords:** grasslands, growing season, nitrogen, precipitation, phenology

## Abstract

Changes in spring and autumn phenology and thus growing season length (GSL) pose great challenges in accurately predicting terrestrial primary productivity. However, how spring and autumn phenology in response to land-use change and nitrogen deposition and underlying mechanisms remain unclear. This study was conducted to explore the GSL and its components [i.e., the beginning of growing season and ending of growing season (EGS)] in response to mowing and nitrogen addition in a temperate steppe on the Mongolia Plateau during 2 years with hydrologically contrasting condition [dry (2014) vs. wet (2015)]. Our results demonstrated that mowing advanced the BGS only by 3.83 days, while nitrogen addition advanced and delayed the BGS and EGS by 2.85 and 3.31 days, respectively, and thus prolonged the GSL by 6.16 days across the two growing seasons from 2014 to 2015. When analyzed by each year, nitrogen addition lengthened the GSL in the dry year (2014), whereas it shortened the GSL in the wet year (2015). Further analyses revealed that the contrasting impacts of nitrogen on the GSL were attributed to monthly precipitation regimes and plant growth rate indicated by the maximum of normalized difference vegetation index (NDV_max_). Moreover, changes in the GSL and its two components had divergent impacts on community productivity. The findings highlight the critical role of precipitation regimes in regulating the responses of spring and autumn phenology to nutrient enrichment and suggest that the relationships of ecosystem productivity with spring and autumn phenology largely depend on interannual precipitation fluctuations under future increased nitrogen deposition scenarios.

## Introduction

Changes in the growing season timing and growing season length (GSL) can regulate biosphere-atmosphere interactions, with consequent carbon (Piao et al., 2007, 2008; Xia et al., 2015) and water cycling (White et al., 1999; Lian et al., 2020; Cheng et al., 2021). The lengthened growing season resulting from advanced beginning of growing season (BGS) and delayed ending of growing season (EGS) has increased primary productivity of terrestrial ecosystems (Piao et al., 2007; Dragoni et al., 2011; Cheng et al., 2021). In addition, carbon loss induced by earlier autumn phenology could counteract the carbon uptake associated with earlier spring phenology, leading to a net carbon loss from terrestrial ecosystems (Piao et al., 2008). Therefore, quantifying the changes in the spring and autumn phenology is critical for the accurate prediction of terrestrial ecosystem carbon balance.

It has been revealed that plant growth could balance from multiple resources (such as nutrient availability) and change allocation to maximize acquisition of the most limiting resources (Bloom et al., 1985; Chapin et al., 1990). This may suggest that elevated anthropogenic nitrogen deposition (Peñuelas et al., 2013; Yu et al., 2019) can alter vegetation activity and thus phenology (Piao et al., 2019, 2020; Vitasse et al., 2021) by providing nitrogen availability. Recent studies have demonstrated that variations in nitrogen or phosphorus availability could change the spring and autumn phenology (Yang et al., 2016; Yin et al., 2016; Fu et al., 2019; Vitasse et al., 2021), as well as the GSL (Wang and Tang, 2019). For example, increased soil nitrogen availability could supplement nutrient deficiencies and thus stimulate plant growth under low temperature in early autumn (McCormack et al., 2014; Delpierre et al., 2016; Yin et al., 2016), which can delay the EGS. In addition, nitrogen addition could also decrease the cellular maturation rate (Kalliokoski et al., 2013; Cuny et al., 2015), and consequently postpone the autumn phenology (Wingler et al., 2006). In nitrogen-limited ecosystems, species usually allocate more resources to growth (LeBauer and Treseder, 2008) under increased nitrogen availability (Xiang et al., 2016), which can delay the reproductive stages (Cleland et al., 2006), thus delaying the senescence date (Wang and Tang, 2019). Nevertheless, whether the effect of nitrogen enrichment on plant phenology regulated by other factors under land-use and climate change scenarios remains largely unknown.

As a widespread land-use practice, mowing has been applied in managed ecosystems (e.g., grasslands; Liu et al., 2017; Zhang et al., 2017) to maintain plant diversity and production. Mowing could affect the microenvironment (such as light, temperature, and moisture) and thus plant growth by removing aboveground litter (Collins et al., 1998; Huhta et al., 2001; Liu et al., 2017). In the early growing season, mowing can elevate soil temperature by increasing light availability and thus accumulated temperature, which is critical for driving plant phenology (Fu et al., 2015; Piao et al., 2019), and consequently advance the BGS. In the late growing season, mowed grasslands may have lower soil water availability due to increased evaporation associated with less litter cover, which can accelerate the EGS (Liu et al., 2016a,b). In addition, it has been proved that mowing may affect reproductive phenology of early flowering species on the Tibetan Plateau (Liu et al., 2017). Therefore, mowing could have the potential to regulate the responses of spring and autumn phenology to nutrient enrichment. However, the direct field experimental evidence and underlying mechanisms remain limited. Given the critical role of soil water availability in mediating plant growth and phenology in grasslands (Körner, 2015; Quan et al., 2019; Zhou et al., 2019, 2022), precipitation also can have the potential regulation on the response of spring and autumn phenology to mowing, nutrient enrichment, and their interactions.

Considering the above knowledge gaps, this study was conducted to explore the effects of nitrogen addition and mowing (annually) on the GSL and its two components (i.e., BGS and EGS) over two contrasting hydrologically growing seasons from 2014 (dry) to 2015 (wet) in a temperate grassland on the Mongolian Plateau. The specific questions we addressed in this study included: (1) How do nitrogen addition and mowing affect the GSL and its two components? (2) How do environmental factors mediate the responses of GSL and its two components to nitrogen addition and mowing?

## Materials and Methods

### Study Site

This study was located in a semiarid steppe in Duolun Restoration Ecology Research Station, Duolun County (42°02' N, 116°07' E, 1324 m a.s.l.), Inner Mongolia, China. Long-term (1961–2018) mean annual temperature and precipitation were 2.1°C and 385.5 mm, respectively (China Meteorological Data Sharing Service System). The long-term mean annual potential evaporation at this experimental site is 1,748 mm (Wang et al., 2021). The sandy soil is classified as Haplic Calcisol (Food and Agriculture Organization of the United Nations), with 62.75 ± 0.04% sand, 20.30 ± 0.01% silt, and 16.95 ± 0.01% clay. The study site has been fenced since 2001 to exclude cattle and sheep grazing. The dominant plant species in this temperate steppe were *Stipa krylovii, Artemisia frigida*, and *Agropyron cristatum* (Miao et al., 2020; Liu et al., 2021b).

### Experimental Design

A factorial design with four treatments (control, mowing, nitrogen addition, and mowing plus nitrogen addition) and five replications for each treatment was employed in this experiment (Liu et al., 2018; Wang et al., 2020, 2021). There were 20 plots (each was 4 × 4 m^2^) arranged by four rows and five columns, and the buffer zone was 1 m between any two plots. To reduce the edge effect, a 0.5 m buffer zone to the edge of each plot was also designed, thus all the measurements were conducted in the 3 × 3 m^2^ core zone. The mowing treatment was conducted in the late August of each year since 2012. All the plants were mowed 5 cm aboveground to simulated hay harvesting, a widely land-use type in many grasslands (Luo et al., 2001; Niu et al., 2010; Liu et al., 2017; Du et al., 2018). The nitrogen treatment (10 g m^−2^ year^−1^) followed the range of airborne nutrient deposition observed in Northern China (Liu et al., 2021a). Nitrogen (N) was added in each nitrogen addition plot using the form of NH_4_NO_3_ at a rate of 100 kg N ha^−1^ in early May of each year since 2013.

### Soil Temperature Measurement

To investigate the effects of temperature on spring and autumn phenology, we measured soil temperature at a depth of 10 cm that was recorded every 2 h using DS 1923 iButton (Maxim Integrated, San Jose, CA, USA) during the growing season (from May to October in each year). Due to not being waterproof, the sensors were sealed with balloons, which had been demonstrated to be an effective way in other systems to avoid direct exposure to precipitation (Lutterschmidt et al., 2006; Kearney et al., 2011). Then, the wrapped sensors were put into soil at a depth of 10 cm.

### Normalized Difference Vegetation Index Measurement and Data Fitting

We calculated normalized difference vegetation index (NDVI) to understand the community development under different treatments and also obtain the spring (BGS) and autumn phenology (EGS), and thus, the GSL (the difference in day of year of the BGS and EGS). Spectral reflectance of a 1 m × 1 m permanent subplot in each plot was measured at 5- to 7-day intervals with a Tetracam Agricultural Digital Camera (ADC, Tetracam Inc., Chatsworth, CA, USA) at cloud-free noon during each growing season from May to October. The camera was held 1 m above each subplot with an iron shelf during each measurement. NDVI was calculated as

$$\begin{array}{l} \text{NDVI }=\;\frac{\text{Reflectance at }775\text{ nm }-\text{ Reflectance at }675\text{ nm}}{\text{Reflectance at }775\text{ nm }+\text{ Reflectance at }675\text{ nm}} \end{array}$$

Then, we fitted NDVI data with a 5-parameter Weibull function using Sigmaplot 14.0 software (Systat Software, Inc., San Jose, CA, USA). BGS, EGS, GSL, as well as the maximum value of NDVI, were extracted using the methods described by Xia et al. (2015).

### Plant Sampling

To examine whether the changes in spring and autumn phenology affect the community productivity, we measured the aboveground net primary productivity (ANPP). One 0.5 m × 0.5 m quadrat was established in each experimental plot in each of the late growing season (May–October) of 2014 and 2015. Then, we clipped living aboveground biomass in each quadrat and separated into species level. Living aboveground biomass of each species was oven-dried at 65°C for 48 h and weighted to determine the dry mass (Guo et al., 2021).

### Data Analysis

First, we calculated the monthly mean value of soil temperature and then averaged them from May to October as growing season mean values. Then, two-way ANOVAs were used to examine the effects of mowing and nitrogen addition and their interactions on the soil temperature, BGS, EGS, GSL, and the maximum of NDVI_max_, as well as the ANPP. The growing season mean values were used to calculate mowing and nitrogen effects on the above variables. Mowing effects were calculated as [(mowing – control)/control] in the unfertilized plots and [(mowing plus nitrogen addition – nitrogen addition)/nitrogen addition] in the fertilized plots. Nitrogen effects were calculated as [(nitrogen addition – control)/control] in the unmowed plots and [(mowing plus nitrogen addition – mowing)/mowing] in the mowed plots. Mowing and nitrogen effects were calculated in each year. The correlations among variables were explored by Pearson's correlation method. Significant differences were evaluated at the 0.05 probability level. Linear regressions were used to explore the relationships of the BGS, EGS, and GSL with soil temperature, precipitation, and ANPP. All analyses were conducted using SAS 8.0 (SAS Institute Inc., Cary, NC, USA). GraphPad Prism 9.0 (GraphPad Inc., San Diego, CA, USA) was used to plot the graphs.

## Results

### Precipitation Patterns and Variations of Soil Temperature Under Different Treatments

**Figure 1:**
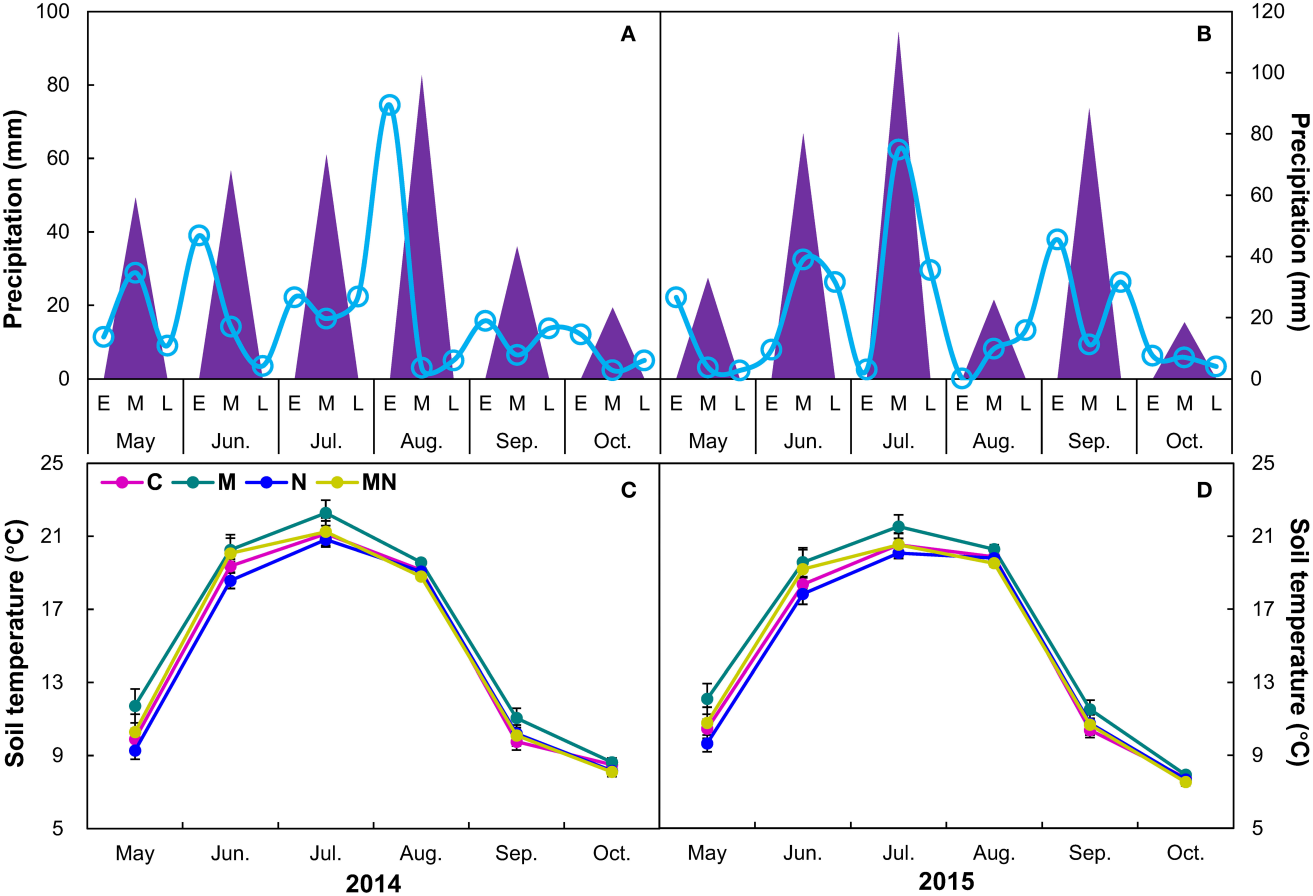
Precipitation (mm) in each month (triangles) and in the early (E), middle (M), late of each month [L, smooth lines **(A,B)**], and effects [Mean ± 1 SE **(C,D)**] of mowing and nitrogen addition on monthly soil temperature during each growing season from 2014 to 2015. C, control; M, mowing; N, nitrogen addition; MN, mowing plus nitrogen addition.

Growing season (May–October) precipitation in 2014 (305 mm) was 12% lower than the long-term mean (346 mm), whereas the precipitation of growing season in 2015 (359 mm) was 3% above the long-term mean. In addition, monthly precipitation fluctuated greater in 2015 than those in 2014 (Figures 1A,B), especially in August. Similarly, precipitation from June to September in 2014 (236 mm) was 21% lower than the long-term mean (299 mm), whereas precipitation from June to September in 2015 (307 mm) was 3% higher than the long-term mean (Figures 1A,B).

No interannual variation of soil temperature was observed over 2 years from 2014 to 2015. Mowing elevated soil temperature by 0.68°C (*p* = 0.04), whereas nitrogen addition had no impact on it over 2 years (Table 1). The effects of mowing on soil temperature did not change with year. There were no interactions between mowing and nitrogen addition on soil temperature (*p* > 0.05, Table 1). When analyzed by each year, both mowing and nitrogen addition had no impact on soil temperature in 2014 or 2015. When analyzed by different stages of growing season, mowing marginally increased soil temperature of the early growing season (May–June) by 1.31°C and 1.33°C (both *p* = 0.08, Supplementary Table S1) in 2014 and 2015, respectively, whereas it had no effects on that of the middle or late growing season in any of 2 years. When analyzed by each month during each growing season, mowing marginally elevated soil temperature only in May by 1.42°C (*p* = 0.08) and 1.38°C (Figures 1C,D, *p* = 0.07, Supplementary Table S2) in 2014 and 2015, respectively. Nitrogen addition had no impact on soil temperature in any of the months in 2 years (Supplementary Table S2).

**Table 1:** Results (*p*-value) of three-way ANOVA on the effects of year, mowing, and nitrogen addition and their interactions on the soil temperature (Soil T), beginning (BGS), ending (EGS), and length of growing season (GSL), as well as the maximum of normalized difference vegetation index (NDVI_max_).

**Source of variation**	**Soil T**	**BGS**	**EGS**	**GSL**	**NDVI_**max**_**
Year	0.822	**<0.001**	**<0.001**	**<0.001**	**<0.001**
Mowing	**0.043**	**0.036**	0.273	0.387	0.928
Nitrogen	0.105	0.051	**<0.001**	**<0.001**	**<0.001**
Year * Mowing	0.975	0.208	0.370	0.200	**0.013**
Year * Nitrogen	0.971	**<0.001**	<0.001	**<0.001**	**<0.001**
Mowing * Nitrogen	0.416	**<0.001**	**<0.001**	**<0.001**	0.529
Year * Mowing * Nitrogen	0.988	**<0.001**	**<0.001**	**<0.001**	0.132

*The bold numbers indicate the significance at p <0.05*.

### Effects of Mowing and Nitrogen Addition on the BGS, EGS, and GSL, as Well as the Maximum of NDVI

**Figure 2:**
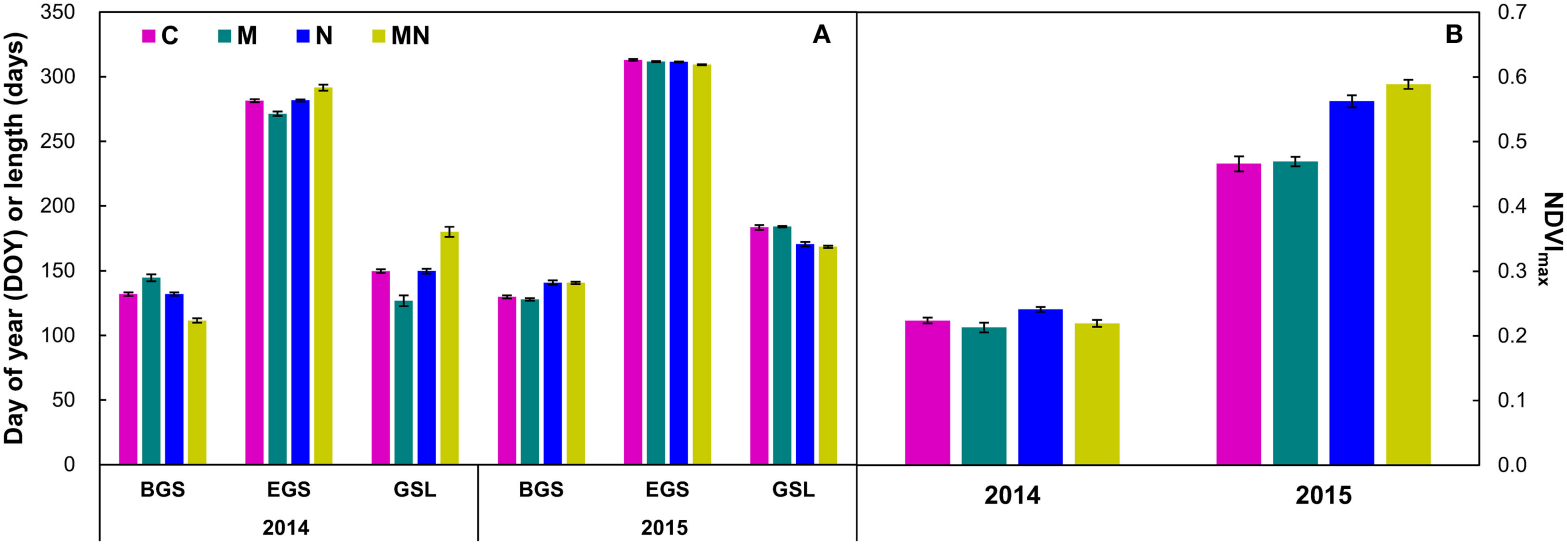
Effects (Mean ± 1 SE) of mowing and nitrogen addition on the beginning (day of year, DOY), ending (DOY), and length of growing season [days **(A)**], as well as the maximum of normalized difference vegetation index **(B)** in 2014 and 2015, respectively. Refer to abbreviations in Table 1 and Figure 1.

Significant interannual variations of BGS, EGS, and GSL, and the maximum of NDVI were found (all *p* < 0.001, Table 1). BGS and EGS of 2014 were 4.74 and 29.87 days earlier than those of 2015, respectively, leading to a shorter growing season in 2014 than that of 2015 (Figure 2A). In addition, the maximum of NDVI in 2014 (0.22 ± 0.00) was also lower than that of 2015 (0.52 ± 0.01, Figure 2B). Over 2 years, mowing advanced the BGS by 2.43 days (*p* = 0.04, Table 2), whereas it had no effects on the EGS or GSL. Nitrogen addition advanced the BGS and delayed the EGS by 2.26 days (*p* = 0.05) and 4.07 days (*p* < 0.001), and thus lengthened the growing season by 6.33 days (*p* < 0.001, Figure 2A). Mowing had no impact on the maximum of NDVI, whereas nitrogen addition increased it by 0.06 (*p* < 0.001, Table 1) over 2 years from 2014 to 2015. Moreover, the effects of nitrogen addition on the BGS, EGS, and GSL, as well as the maximum of NDVI significantly changed with year (all *p* < 0.001).

**Table 2:** Results (*p*-value) of two-way ANOVA on the effects of mowing, nitrogen addition, and their interactions on the BGS, EGS, GSL, and NDVI_max_ in 2014 and 2015.

**Year**	**Source of variation**	**BGS**	**EGS**	**GSL**	**NDVI_**max**_**
2014	Mowing	0.055	0.916	0.249	**0.009**
	Nitrogen	**<0.001**	**<0.001**	**<0.001**	**0.045**
	Mowing*Nitrogen	**<0.001**	**<0.001**	**<0.001**	0.337
2015	Mowing	0.421	**0.003**	0.602	0.171
	Nitrogen	**<0.001**	**<0.001**	**<0.001**	**<0.001**
	Mowing*Nitrogen	0.505	0.396	0.371	0.237

*Refer to abbreviations in Table 1*.

*The bold numbers indicate the significance at p <0.05*.

When analyzed by each year, mowing marginally advanced the BGS by 3.85 days (*p* = 0.06, Table 2), whereas it had no impact on the EGS or GSL, whereas nitrogen addition advanced the BGS and delayed the EGS by 16.54 and 10.20 days, respectively, and thus extended the growing season by 26.73 days in 2014 (Figure 2A, all *p* < 0.001, Table 2). Mowing only advanced the EGS by 1.73 days in 2015 (*p* < 0.01). In contrast to 2014, nitrogen addition delayed the BGS and advanced the EGS by 12.02 and 2.05 days, respectively, leading to a shortened growing season (Figure 2A, all *p* < 0.001, Table 2). Mowing decreased, whereas nitrogen addition elevated the maximum of NDVI by 0.02 (*p* = 0.04) and 0.01 (*p* < 0.01, Table 2), respectively, in 2014. Mowing had no impact on the maximum of NDVI (*p* > 0.05), while nitrogen addition increased it by 0.11 (*p* < 0.001) in 2015.

### Relationships of the BGS, EGS, and GSL, as Well as the Maximum of NDVI With Soil Temperature and Precipitation in Different Stages of Growing Season

**Figure 3:**
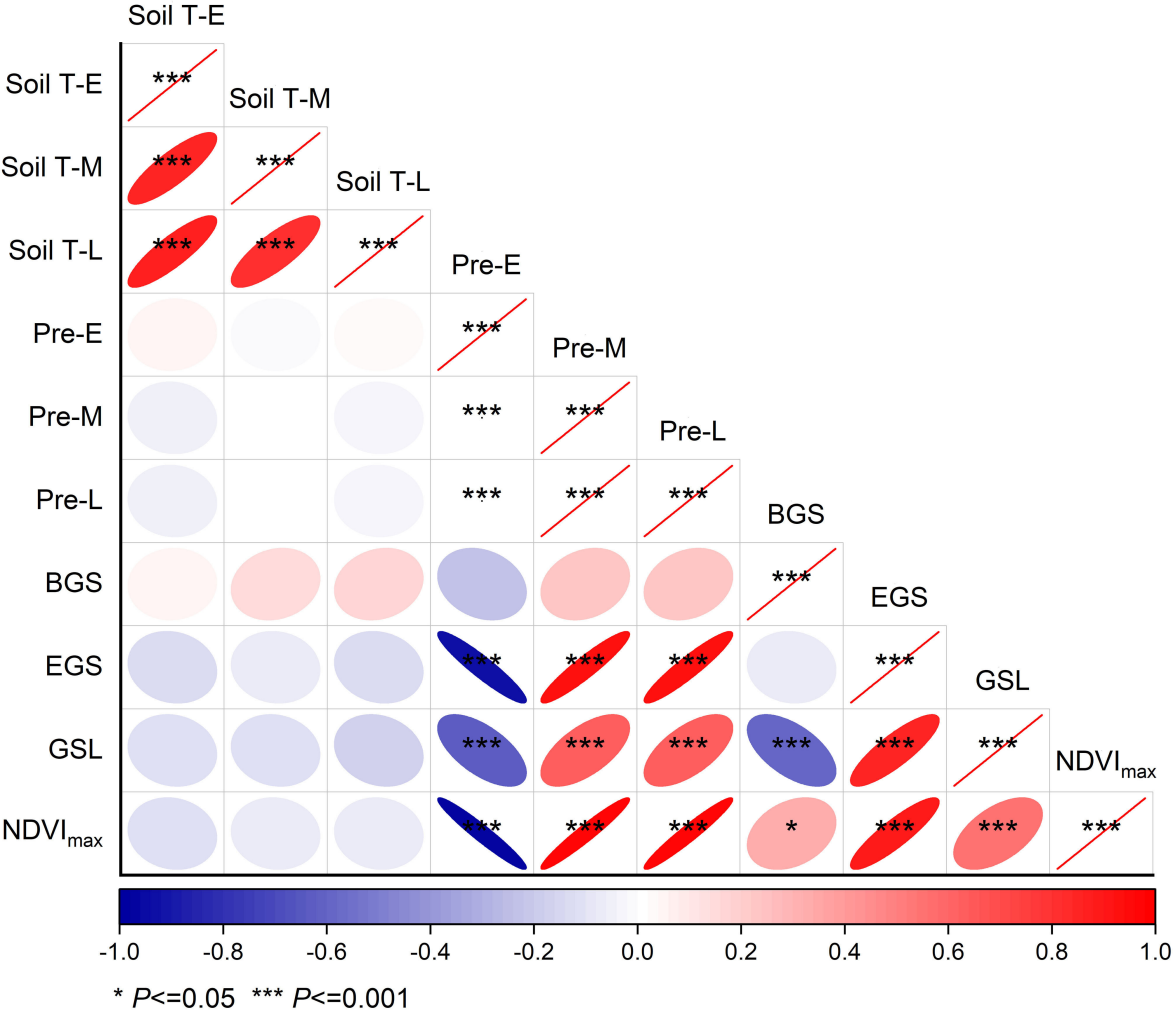
Relationships of beginning, ending, and length of growing season, as well as the maximum of normalized difference vegetation index with soil temperature and precipitation in the early (E, May–June), middle (M, July–August), and late (L, September–October) of growing season, respectively, over 2 years from 2014 to 2015. Refer to abbreviations in Table 1.

Our results showed that there were no relationships of the beginning, ending, and length of growing season, and the maximum of normalized difference index with soil temperature in any stages of growing season across the 2 years (Figure 3). When analyzed by each month, the results revealed positive relationships of EGS and the maximum of NDVI with soil temperature in August (Supplementary Figure S1). In contrast, negative dependences of ending and length of growing season, as well as the maximum of NDVI on soil temperature in October were observed (Supplementary Figure S1).

In addition, EGS showed negative dependence on early growing season precipitation, whereas positive dependence on middle and late growing season precipitation. Similar relationships of GSL and the maximum of NDVI with middle and late growing season precipitation were also found, respectively, over the 2 years from 2014 to 2015 (Figure 3). When analyzed by each month, there were no relationships of BGS with precipitation of any month (Supplementary Figure S2). EGS showed negative relationships with precipitation in May, June, and October, whereas positive relationships with precipitation in July, August, and September (Supplementary Figure S2). Similar patterns were also found in GSL and the maximum of NDVI.

### Impacts of Changes in the BGS, EGS, and GSL, as Well as the Maximum of NDVI on Aboveground Net Primary Productivity

**Figure 4:**
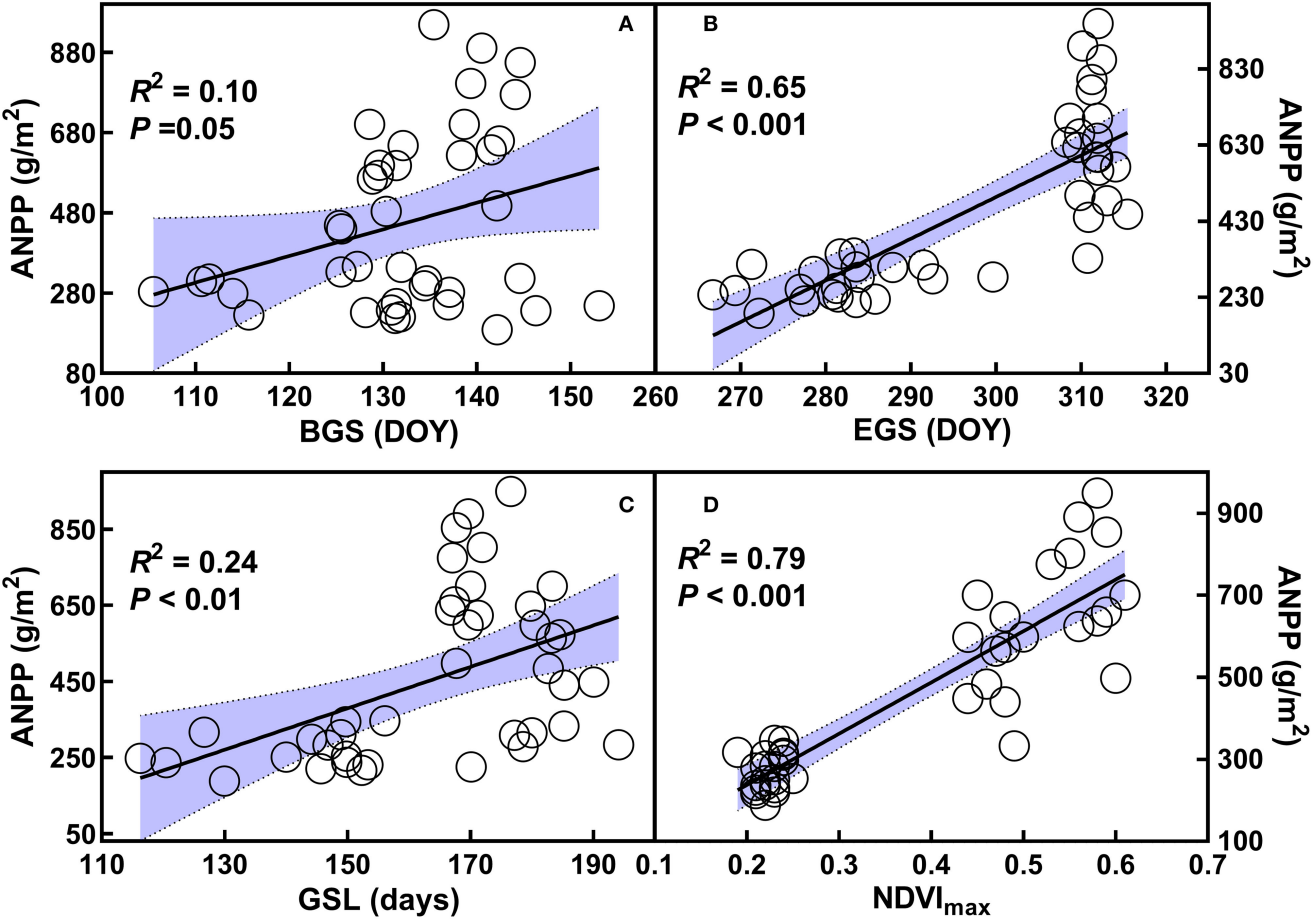
Relationships of aboveground net primary productivity (ANPP) with beginning **(A)**, ending **(B)**, and length of growing season **(C)**, as well as the maximum of normalized difference vegetation index **(D)** over the two years from 2014 to 2015. Refer to abbreviations in Table 1.

Positive dependences of ANPP on BGS (*R*^2^ = 0.10, *p* = 0.01), EGS (*R*^2^ = 0.65, *P* < 0.001), GSL (*R*^2^ = 0.24, *p* < 0.01), and the maximum of NDVI (*R*^2^ = 0.79, *p* < 0.001) were found over 2 years from 2014 to 2015 (Figures 4A–D). When analyzed by each year, there were no dependences of ANPP on the BGS, EGS, or GSL, or the maximum of NDVI in 2014 (Supplementary Figures S4a–d). In contrast, positive relationships of ANPP with the BGS (*R*^2^ = 0.41, *p* < 0.01) and the maximum of NDVI (*R*^2^ = 0.27, *p* = 0.02) were found in 2015 (Supplementary Figures S5a,d). Surprisingly, ANPP showed negative dependence on the GSL in 2015 (*R*^2^ = 0.31, *p* = 0.01, Supplementary Figure S5c).

## Discussion

### Effects of Mowing on GSL and Its Two Components

It has been well-documented that temperature plays a critical role in regulating spring and autumn phenology, and thus GSL in terrestrial ecosystems (Cleland et al., 2007; Peñuelas et al., 2009; Chuine et al., 2010). For example, spring temperature drives the onset of spring phenology in the northern hemisphere (Piao et al., 2007, 2015). In addition, higher temperatures could delay autumn phenology in temperate China (Piao et al., 2007; Liu et al., 2016a). As a result, increased temperature could lengthen the growing season by advancing the spring phenology and delaying the autumn phenology. In this study, although mowing increased soil temperature and advanced the BGS over the two growing seasons, no robust relationships of BGS with temperature were found (Figure 3, Supplementary Figure S1). In fact, recent studies have shown that the control role of temperature in mediating spring phenology is declining (Fu et al., 2015), which can partly support the weak relationships of GSL and its two components with soil temperature in this study. This observation also indicates that other factors (except for temperature) can mediate spring phenology under mowing.

Photoperiod has been demonstrated to affect plant phenology in terrestrial ecosystems (Körner and Basler, 2010; Flynn and Wolkovich, 2018). Longer photoperiod can advance the spring phenology of tree species (Chuine et al., 2010). In this study, by removing the standing litter, mowing could also increase the light availability for the short species (especially for *Potentilla acaulis* L., an early species in the study site) in the early growing season, during which the short species can have the potential to accelerate growth and thus advance the BGS across 2 years. In addition, leaf unfolding could be also regulated by the height-time hypothesis that short individuals can advance their leaf unfolding date (Sun and Frelich, 2011; Liu et al., 2021a). In this study, mowing indeed decreased plant height in the early growing season (unpublished data), which can consequently advance the spring phenology. Nevertheless, when analyzed by each year, the advancement effects of mowing were not significant, especially in 2015, which may be attributed to the insufficient soil water availability due to increased light availability and thus evaporation under mowing. Given the important role of soil water availability in regulating spring phenology in temperate steppes (Shen et al., 2015; Luo et al., 2021), the deficient available water in the soil may weaken the stimulated temperature and light availability associated with mowing in this study. In addition, the findings of mowing did not affect the EGS, suggesting that autumn phenology might not be sensitive to changes in microclimate factors associated with mowing in temperate grasslands. Moreover, given that changes in photoperiod could affect the response of plant phenology to temperature (Basler and Körner, 2014; Way and Montgomery, 2015), the increased light availability under mowing in the early growing season may also interact with temperature to affect spring phenology in the temperate grassland. Further manipulative evidence is still needed to support the assertions.

### Effects of Nitrogen Addition on GSL and Its Two Components Depend on Precipitation

Nitrogen is one of the most important factors affecting plant growth and phenology (Jing et al., 2017; Liu et al., 2018; Vitasse et al., 2021), especially in nitrogen-limited ecosystems (such as grasslands). Nitrogen addition could stimulate plant growth and thus phenology (Fu et al., 2019; Wang and Tang, 2019). A meta-analysis has shown that nitrogen addition advances leaf senescence across all the biomes, including forest, grassland, cropland, and desert (Wang and Tang, 2019). Surprisingly, the effects of nitrogen addition on the GSL and its two components were opposite in the two hydrologically contrasting growing seasons in this study. In the dry growing season (2014), nitrogen addition advanced the BGS and delayed the EGS, leading to a lengthened growing season (Figure 2A). In contrast, nitrogen addition delayed the BGS and advanced the EGS, resulting in a shortened growing season (Figure 2A) in the wet growing season (2015). The contrasting findings are similar to those found in a previous study, which demonstrates the opposite phenological response in dry vs. wet years (Bao et al., 2021). Normalized difference vegetation index can be used an index of photosynthesis in ecosystems with low leaf area index or vegetation cover (Wohlfahrt et al., 2010; Del Grosso et al., 2018). In the dry growing season, plants had a lower growth rate indexed as the maximum of NDVI compared with those in the wet growing season (Figure 2B). The low growth rate of plants could reduce nitrogen use, which can result in retaining more nitrogen in the soil and subsequently used by plants when small precipitation events occur. In addition, precipitation in the early growing season is critical for driving the spring phenology (Shen et al., 2015; Ganjurjav et al., 2020; Wang et al., 2022). In this study, precipitation in May of 2014 was 40% greater than that of long-term mean (Supplementary Figure S3), which provides water requirement for plants to begin to grow in the early growing season (Shen et al., 2015; Luo et al., 2021). Along with the favorable precipitation condition, addition of nitrogen could further accelerate plant growth and consequently advance the BGS in 2014. In addition, relatively low values of the maximum of NDVI associated with lack of precipitation in the middle of the growing season could decrease the nitrogen use. Lower precipitation could also be favorable for retaining nitrogen into soil without being leached, which allows plants to use nitrogen more efficiently when precipitation events occur. In fact, small but steady precipitation indeed occurs in the late growing season of 2014, which stimulates the nitrogen effects on maintaining plant growth in the late growing season and thus delays the EGS, consequently lengthening the growing season in 2014. The relationships of the EGS and GSL with monthly precipitation (Figure 3, Supplementary Figure S2) support the above arguments.

In contrast, most of the precipitation in May occurred in the early stage of 2015 (Figure 1B, Supplementary Figure S3) and could not supply steady and consecutive water conditions in the middle and late May for plants to begin to grow. Therefore, the less precipitation in the early growing season might weaken the nitrogen effects on plant growth, which led to the delayed BGS in 2015 (Shen et al., 2015; Ganjurjav et al., 2020; Wang et al., 2022). In addition, although precipitation of 2015 was greater than that of the long-term mean, most of the precipitation occurred in June, July, and September (Figure 1B). On the one hand, high precipitation in the middle growing season (except for precipitation in August) significantly stimulated plant growth (indicated by the maximum of NDVI, Figure 2B) and thus for nitrogen use. On the other hand, high precipitation in the middle growing season could also increase nitrogen leaching (Brandt et al., 2010), which can lead to decreased soil-available nitrogen in the late growing season. Moreover, precipitation in the late growing season, especially in October, was low compared with that in 2014 (Figures 2A,B). The low soil-available nitrogen combined with low precipitation was not favorable for plant growth and resulted in accelerated leaf senescence in the late growing season (Liu et al., 2016a,b; Ren and Peichl, 2021). Thus, the delayed BGS and advanced EGS under nitrogen addition shortened the growing season in the wet growing season in 2015. Our findings indicate that precipitation plays a considerable role in regulating the nutrient effects on spring and autumn phenology in temperate grasslands. Given the diverse driving factors for plant phenology (Piao et al., 2019), the effects of other factors (except for precipitation) on spring and autumn phenology are still needed to be investigated in the future (Zhou et al., 2022).

### Interactive Effects of Mowing and Nitrogen Addition on GSL and Its Two Components

To the best of our knowledge, our observations of interaction between mowing and nitrogen addition on spring and autumn phenology, as well as GSL, provide the first experimental evidence on the phenological responses under mowing and nitrogen enrichment in temperate steppes. However, the interactions between mowing and nitrogen addition were different in the two hydrologically contrasting years. The interactive effects of mowing and nitrogen addition on GSL and its two components observed in this study could be largely ascribed to those in 2014 (Tables 1, 2). We found that nitrogen addition had no impact on GSL and its two components without mowing, whereas advanced and delayed spring and autumn phenology with mowing, respectively, and thus extended GSL in 2014. Given that grassland in this study site is nitrogen limitation, nitrogen addition can stimulate plant growth and standing litter accumulation (Liu et al., 2018), which may decrease light availability and temperature accumulation and thus have negative effects on leaf unfolding of short species in the next growing season (Piao et al., 2015; Beil et al., 2021). Mowing can weaken the above negative effects by removing standing litter and thus providing light availability and temperature requirements for driving plant phenology (Flynn and Wolkovich, 2018; Piao et al., 2019), and consequently have substantial impacts on spring and autumn phenology, as well as GSL. Because of low precipitation in 2014, the accumulation of standing litter is less than that in 2013, which cannot be enough to cause the light and temperature limitation in 2015. As a consequence, no interactive effects of mowing and nitrogen addition on GSL and its two components were observed in 2015. These findings suggest that interactions between mowing and nutrient addition could also be mediated by precipitation regimes in different years. Given the changing precipitation regimes, including amount, frequency, intensity, and temporal distributions (IPCC, 2018), it is needed to conduct multifactor manipulative experiments to better understand the realistic response of vegetative phenology to global change.

### Implications for Community Productivity in the Temperate Steppe

GSL and its components have critical roles in affecting ecosystem productivity and its interannual variation (Piao et al., 2007, 2008; Xia et al., 2015); however, their roles remain unclear in the grassland ecosystems, especially under land-use change and nitrogen deposition scenarios. In this study, ANPP in 2015 is greater than that in 2014; precipitation could be one of the limiting factors for the lower ANPP in 2014. In addition to precipitation, our observations of the relationships of ANPP with BGS, EGS, and GSL, as well as the maximum of NDVI (Figure 4), suggest that GSL and growth rate are also considerable factors in mediating ANPP in the temperate steppe. Nevertheless, the negative dependence of ANPP with GSL is not consistent with that reported by previous studies (White et al., 1999; Wu et al., 2012; Michaletz et al., 2014), which have shown that longer GSL generally increase ecosystem productivity. In fact, the negative relationship between ANPP and GSL can be affected by the nitrogen addition. The shortened growing season resulting from delaying the BGS and advancing the EGS combined by increasing maximum of NDVI in 2015 under nitrogen addition could explain the above findings. The positive effects of increasing the maximum of NDVI on ANPP (Supplementary Figure S5) under nitrogen addition can offset the negative effects of shortened growing season and consequently enhance ANPP in 2015. This indicates that the maximum of growth rate could determine the ecosystem productivity irrespective of shortened GSL. However, we did not observe the relationships of ANPP with GSL and its components in 2014, which indicate that low precipitation amount may affect the relationships among them. The findings highlight the jointly control roles of GSL and growth rate in mediating ecosystem productivity in temperate steppes under land-use change and increased nitrogen deposition scenarios.

### Conclusion

Using a 2-year field manipulative experiment with mowing and nitrogen addition, we demonstrated that nitrogen addition showed divergent impacts on GSL and its two components (i.e., BGS and EGS), that is, nitrogen addition advanced the BGS and delayed the EGS in the dry year, whereas it delayed the BGS and advanced the EGS in the wet year. In addition, the effect of nitrogen addition on the maximum of NDVI was larger in the wet year than that in the dry year, indicating that the impacts of nitrogen on vegetation activity were dependent on precipitation regimes. The changes in the GSL and the maximum of NDVI had also diverse impacts on ANPP in years with different precipitation, suggesting that precipitation can enhance the dependences of ecosystem productivity on spring and autumn phenology under nitrogen deposition scenarios in the temperate steppe. The findings promote our understanding on the effects of land-use change and nitrogen deposition on vegetation activity and productivity among years with different precipitation regimes.

## Data Availability Statement

The original contributions presented in the study are included in the article/Supplementary Material, further inquiries can be directed to the corresponding author/s.

## Author Contributions

ZZ, YL, and YM originally formulated the idea. KZ, WW, and JZ developed methodology. SC and HZ conducted fieldwork. ZZ, LZ, and YL generated data analyses, and wrote and revised the manuscript. All authors contributed to the article and approved the submitted version.

## Funding

This study was financially supported by the National Natural Science Foundation of China (42107225, 31670477, 31200375) and the Postdoctoral Innovation and Practice Base of Anyang Institute of Technology (BSJ2020021, BHJ2021007).

## Conflict of Interest

The authors declare that the research was conducted in the absence of any commercial or financial relationships that could be construed as a potential conflict of interest.

## Publisher's Note

All claims expressed in this article are solely those of the authors and do not necessarily represent those of their affiliated organizations, or those of the publisher, the editors and the reviewers. Any product that may be evaluated in this article, or claim that may be made by its manufacturer, is not guaranteed or endorsed by the publisher.
