# Supplementary material for: Contrasting Effects of Nitrogen Addition on Vegetative Phenology in Dry and Wet Years in a Temperate Steppe on the Mongolian Plateau
Source: Front Plant Sci. 2022 Apr 25;13:861794. doi: 10.3389/fpls.2022.861794 (PMC9083225; doi:10.3389/fpls.2022.861794)

**SUPPLEMENTAL MATERIALS**

**Title:** Precipitation regimes regulate fertilization impacts on spring and autumn phenology in a temperate steppe on the Mongolia Plateau

**Authors:** Zhenxing Zhou^1,2,3,†^, Liwei Zhang^1,†^, Yinzhan Liu^1^, Kunpeng Zhang^2^, Wenrui Wang^1^, Junkang Zhu^1^, Shijie Chai^1^, Huiying Zhang^1^, Yuan Miao^1,*^

^1^International Joint Research Laboratory for Global Change Ecology, School of Life Sciences, Henan University, Kaifeng, Henan, China

^2^School of Biological and Food Engineering, Anyang Institute of Technology, Anyang, Henan, China

^3^Taihang Mountain Forest Pests Observation and Research Station of Henan Province, Linzhou, Henan, China

^†^These authors have contributed equally to this work and share first authorship.

^*^**Correspondence author:** Yuan Miao, e-mail: [miaoyuan0921@126.com](mailto:miaoyuan0921@126.com).

**Table S1.** Results (*P*-value) of 2-way ANOVA on the effects of mowing and nitrogen addition on the growing-season mean, early, middle, and late of growing season soil temperature from 2014 to 2015.

| Source of variation | 2014 | | | |  | 2015 | | | |
| --- | --- | --- | --- | --- | --- | --- | --- | --- | --- |
|  | Mean | Early | Middle | Late |  | Mean | Early | Middle | Late |
| Mowing | 0.155 | 0.082 | 0.309 | 0.359 |  | 0.158 | 0.078 | 0.340 | 0.375 |
| Nitrogen | 0.251 | 0.295 | 0.200 | 0.334 |  | 0.261 | 0.301 | 0.175 | 0.436 |
| Mowing*Nitrogen | 0.565 | 0.945 | 0.400 | 0.258 |  | 0.571 | 0.900 | 0.458 | 0.221 |

**Table S2.** Results (*P*-value) of 2-way ANOVA on the effects of mowing and nitrogen addition on the monthly-mean soil temperature in each growing season from 2014 to 2015.

| Year | Source of variation | May | Jun. | Jul. | Aug. | Sep. | Oct. |
| --- | --- | --- | --- | --- | --- | --- | --- |
| 2014 | Mowing | 0.084 | 0.095 | 0.245 | 0.798 | 0.232 | 0.889 |
|  | Nitrogen | 0.200 | 0.472 | 0.318 | 0.160 | 0.607 | 0.123 |
|  | Mowing*Nitrogen | 0.608 | 0.657 | 0.592 | 0.241 | 0.167 | 0.714 |
| 2015 | Mowing | 0.065 | 0.102 | 0.225 | 0.820 | 0.253 | 0.959 |
|  | Nitrogen | 0.149 | 0.547 | 0.226 | 0.164 | 0.613 | 0.225 |
|  | Mowing*Nitrogen | 0.719 | 0.921 | 0.645 | 0.252 | 0.197 | 0.427 |

**Figure S1.** Relationships of the beginning (BGS), ending (EGS), and length of growing season (GSL), as well as the maximum of normalized difference vegetation index (NDVI_max_) with monthly soil temperature (May to October) over the 2 years from 2014 to 2015.


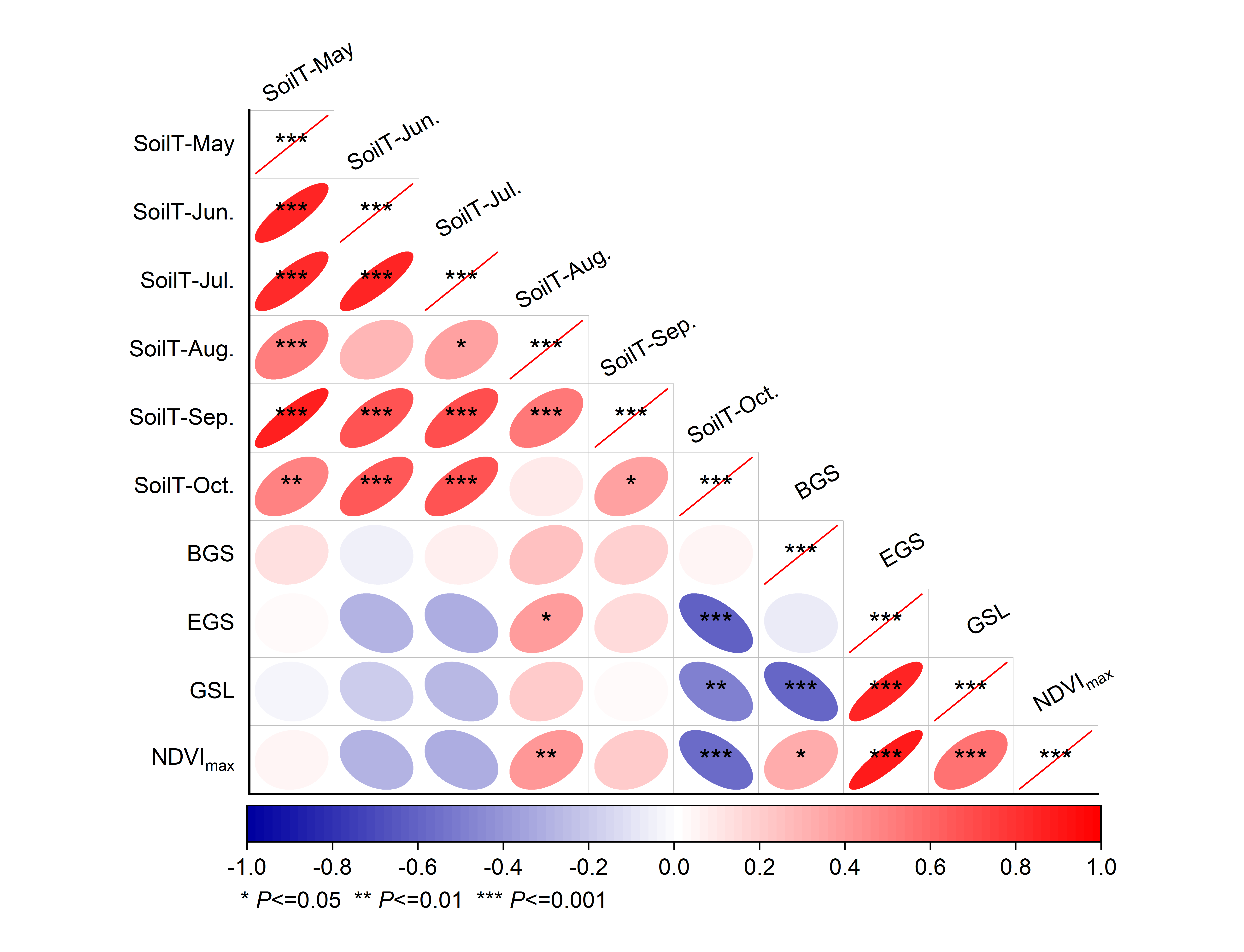


**Figure S2.** Relationships of the BGS, EGS, and GSL, as well as the NDVI_max_ with monthly precipitation (May to October) over the 2 years from 2014 to 2015.


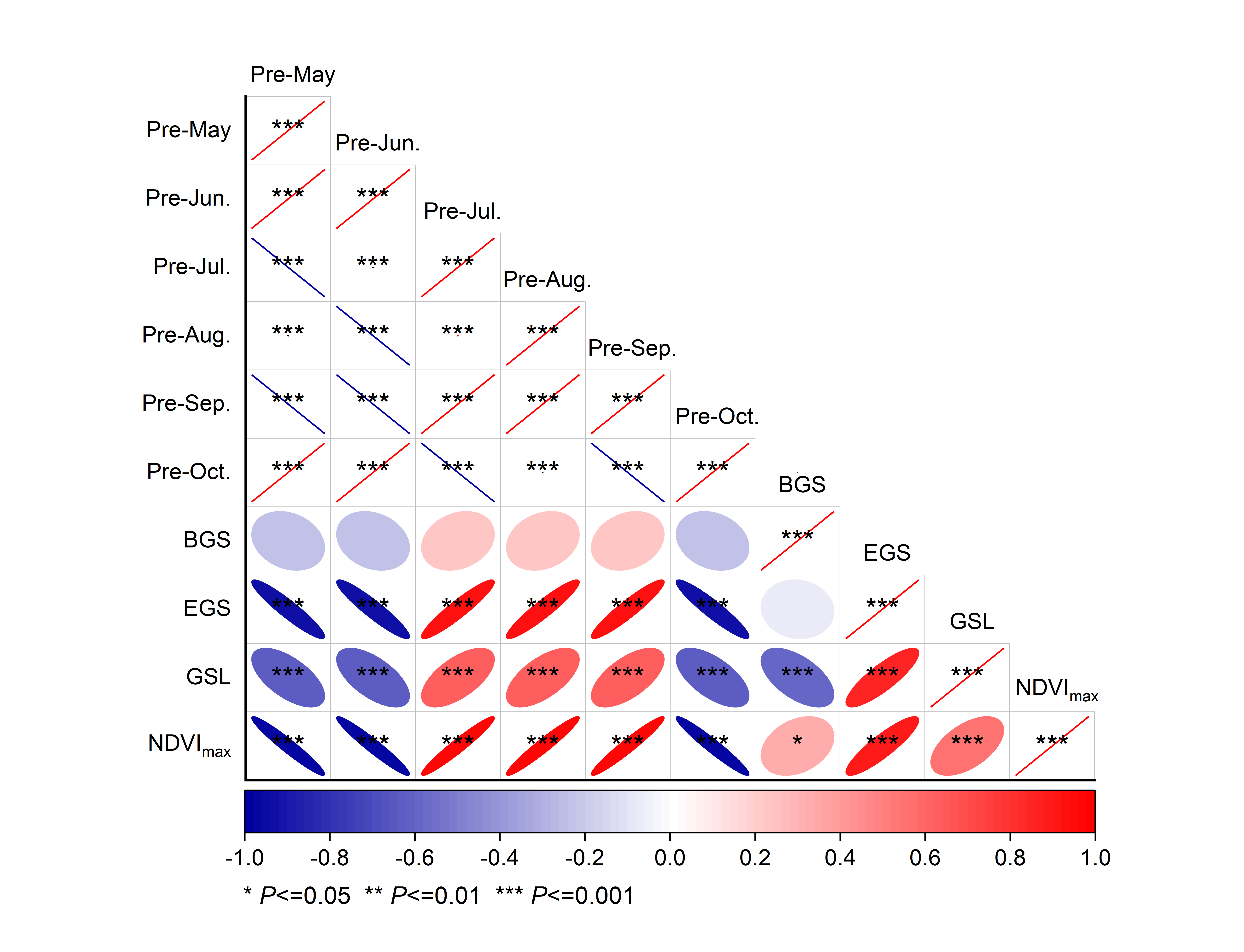


**Figure S3.** Precipitation in May of 2014 and 2015.

**Figure S4.** Relationships of ANPP with BGS (a), EGS (b), GSL (c), as well as NDVI_max_ (d) in 2014. See abbreviations in Figure S1.


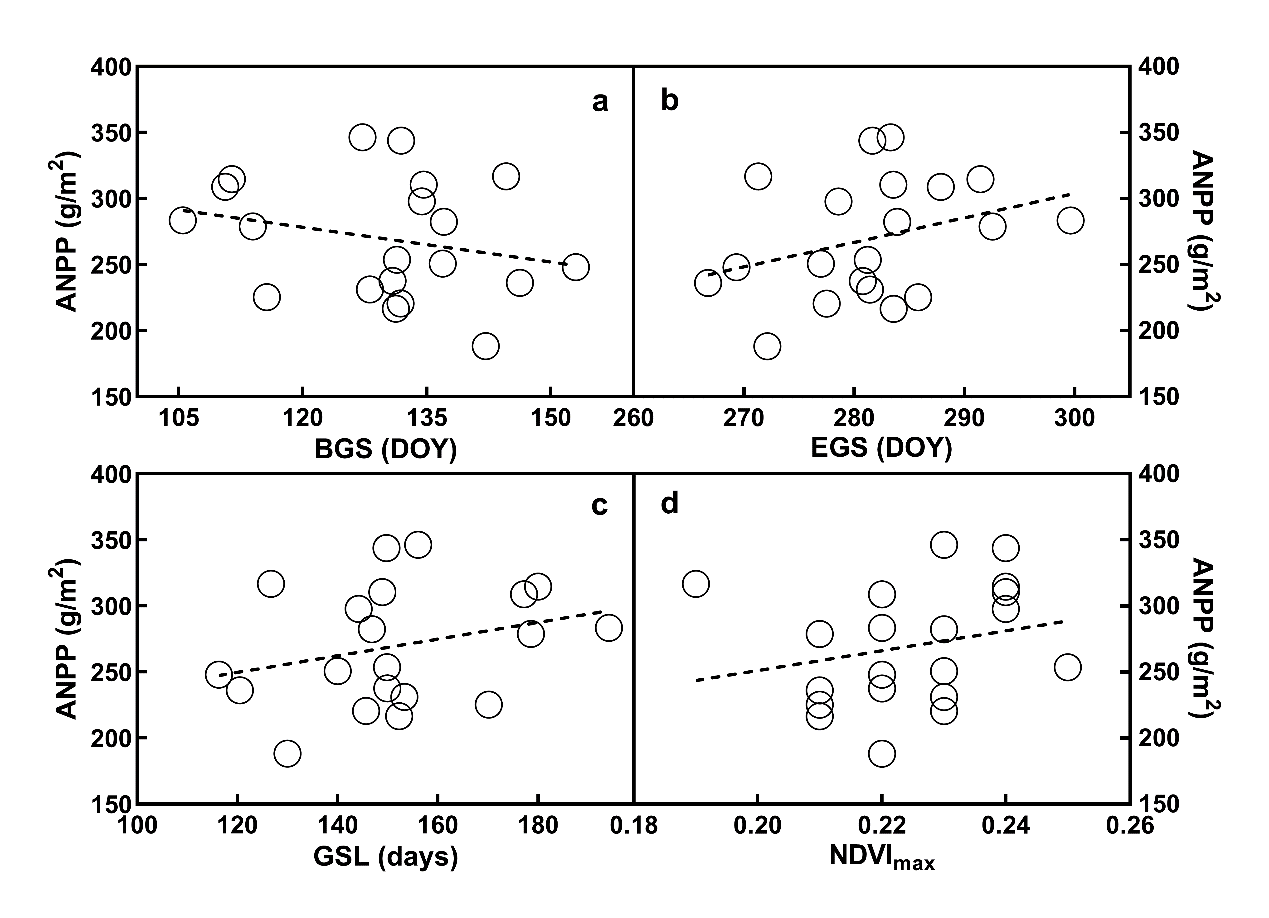


**Figure S5.** Relationships of ANPP with BGS (a), EGS (b), GSL (c), as well as NDVI_max_ (d) in 2015. See abbreviations in Figure S1.


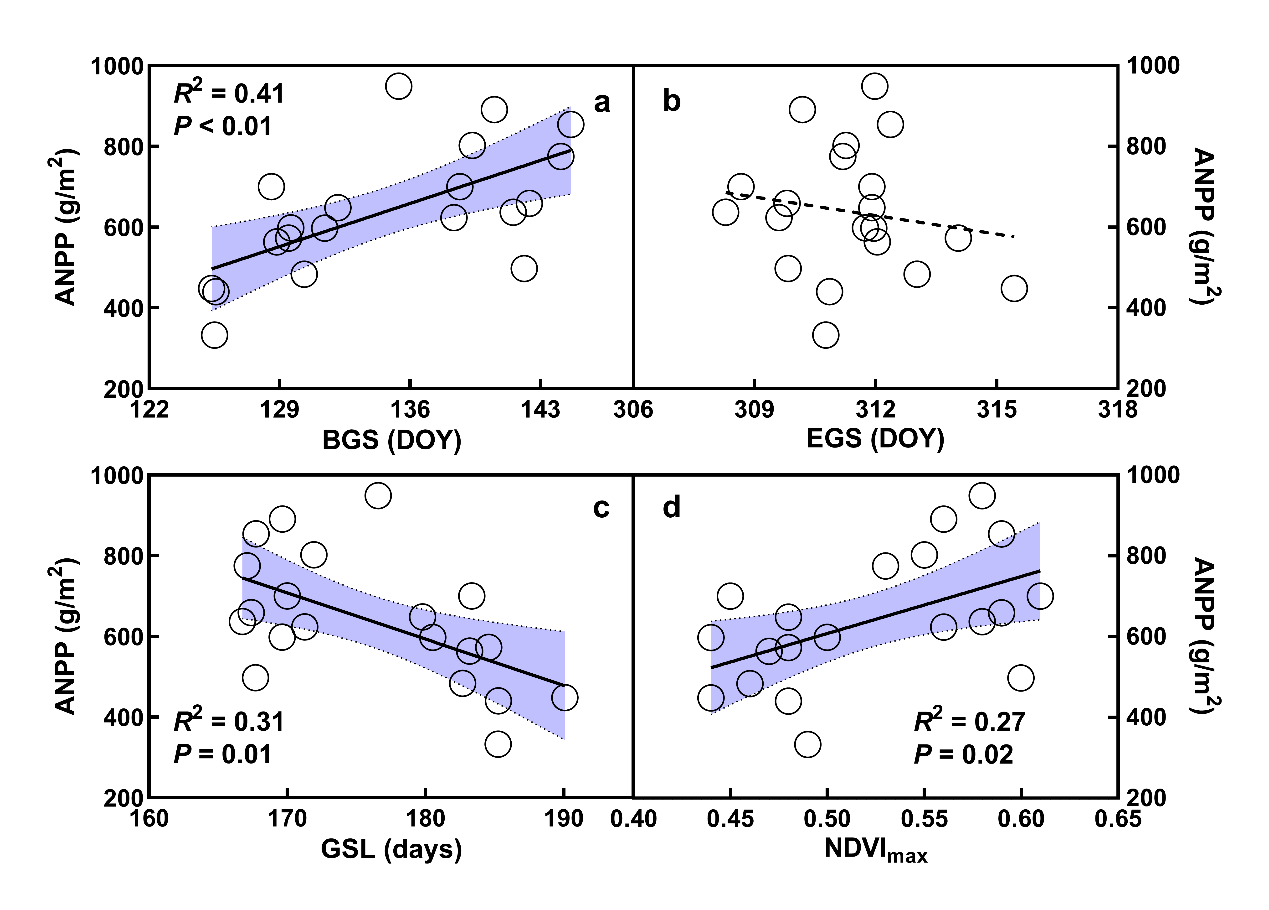

Supplement: Supplementary file 1 [file Data_Sheet_1.docx]
